# Importance of Autophagy in Mediating Cellular Responses to Iron Overload in Cardiomyocytes

**DOI:** 10.31083/j.rcm2305167

**Published:** 2022-05-07

**Authors:** Eddie Tam, Chloe Reno, Khang Nguyen, Sungji Cho, Gary Sweeney

**Affiliations:** ^1^Department of Biology, York University, Toronto, ON M3J 1P3, Canada

**Keywords:** iron overload, autophagy, adiponectin, oxidative stress, endoplasmic reticulum stress, mitochondria, ferroptosis

## Abstract

Both iron overload and deficiency can promote development of cardiomyopathy. Advances in our knowledge from recent research have indicated numerous potential cellular mechanisms. Regulation of myocardial autophagy by iron is of particular interest and will be reviewed here. Autophagy is already well established to play a significant role in regulating the development of heart failure. This review will focus on regulation of autophagy by iron, crosstalk between autophagy and other cellular process which have also already been implicated in heart failure (oxidative stress, mitochondrial dysfunction, endoplasmic reticulum stress, ferroptosis) and the therapeutic potential of targeting these interactions.

## 1. Autophagy: Physiological Function and Impact on Heart Failure

Autophagy is a highly conserved multi-step process which results in the lysosomal degradation of damaged or dysfunctional intracellular components [1]. This is a crucial process especially during stress. For instance, under stressors such as nutrient starvation, ischemia/reperfusion injury or pathogenic infection, various cell types rely on the upregulation of autophagy to abate the harmful consequences of such events [2]. Without this safety mechanism properly in place, a variety of biological processes can devolve. Indeed, autophagy deficiency or dysregulation has now been implicated in several pathological conditions, including heart failure, neurodegenerative diseases, and diabetes [3, 4].

Autophagy is initiated with the formation of the autophagosome which mediates vesicular transport of target cargo prior to lysosomal degradation [5]. During the autophagy process, microtubule-associated protein 1A/1B-light chain 3 (LC3) is recruited to autophagosome membranes, and the conversion of LC3 into its lipidated form (LC3-II) is a marker for detecting autophagosome accumulation. P62 is a generic cargo adapter protein which is degraded upon autophagosome fusion with the lysosome [6]. Together with LC3-II, P62 is considered a reliable marker for measuring the rate of autophagic degradation (also known as autophagic flux).

Autophagy has been described as a “double-edged sword”, characterized both by its cardio-protective and its detrimental properties [4]. On one hand, it serves as a compensatory mechanism that promotes the capture and clearance of damaged or toxic cytoplasmic substances through lysosomal degradation and recycling [5]. It also ensures proper intracellular quality control by limiting the accumulation of misfolded proteins and mitochondrial malfunction. These functions reduce the risk of myocardial damage. On the other hand, excessive or prolonged autophagy may contribute to myocardial damage as it leads to increased cell death [6].

Within the myocardium, autophagy plays a crucial role in cardiac remodelling immediately following injury [7]. According to previous studies, loss of autophagy may lead to cardiomyopathy. For example, in a study by Nakai *et al*. [8], it was found that deficiency in ATG5 (a required protein for autophagy) within the heart led to contractile dysfunction and cardiac hypertrophy. ATG5 deficiency in the heart did not result in changes in fibrosis or myofibrillar disarray [7]. Similarly, Ma *et al*. [9] found that a reduction in beclin-1 promoted autophagy and protected against hypoxia-reoxygenation induced cell death in cardiomyocytes by inhibiting mTOR, reducing mitochondrial depolarization, and activating the transcription factor EB/peroxisome proliferator-activated receptor gamma coactivator 1-alpha signaling axis. Lastly, a study by Gupta *et al*. [10] demonstrated that, UBC9 (a ubiquitin conjugating enzyme) can mediate SUMOylation, which is known to promote autophagy in the heart. According to Gupta *et al*. [10], overexpression of UBC9 protects against cardiac proteotoxicity through increased autophagy [10].

**Fig. 1.**
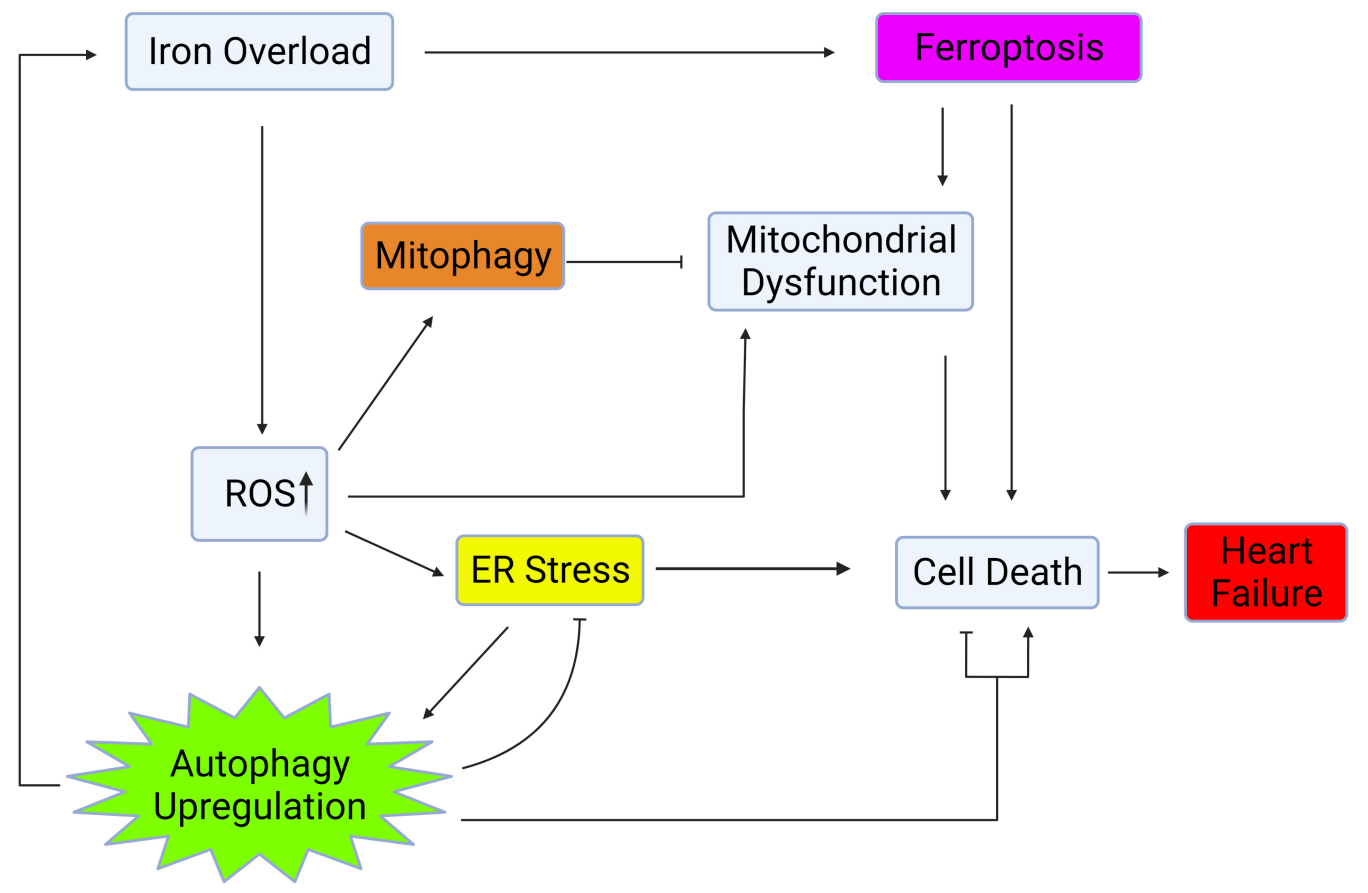
**Autophagy regulation via crosstalk with mitochondrial health, ER stress, and oxidative stress**. Iron overload causes cell death and heart failure through activation of ferroptosis, upregulation of ROS, ER stress, and mitochondrial dysfunction. Upregulation of autophagy and mitophagy plays a role in mitigating the deleterious effects of iron overload within the heart. Created with BioRender.com.

Autophagy is regulated by several factors such as nutrient availability, metabolic signals, and via crosstalk with other cellular processes including ER stress, mitochondrial health, and oxidative stress [11, 12, 13, 14, 15, 16] as shown in Fig. 1. This review will further explore the influence of iron on factors which regulate and crosstalk with autophagy and the significance of these interactions on heart failure.

## 2. Temporal Alterations in Autophagy in Response to Iron

Iron is of considerable interest due to recent studies that have revealed it to be a potent regulator of autophagy [17]. Moreover, iron overload can occur clinically for various reasons including genetic defects, chronic hepatitis C infection, liver disease, iron supplements or blood transfusions. The association between iron deficiency and cardiomyopathy has been well characterized [18]. Moreover, iron deficiency has been recognized as a co-morbidity in 37–61% of patients suffering from chronic heart failure [19]. In contrast, the involvement of iron overload (IO) has also been established but less well known [20]. IO occurs when intracellular ferrous iron (${\mathrm{Fe}}^{2+}$) levels saturate endogenous binding proteins, principally ferritin, and the excess iron forms a labile pool. In the unbound form, iron is highly reactive due to its redox capabilities, and therefore excess iron has various metabolic implications, including the generation of reactive oxygen species (ROS). Emerging reports have identified IO in several pathological conditions, such as polyneuropathy, myopathy, metabolic syndrome, and various cardiovascular diseases (CVDs) [21, 22]. In fact, mortality rates in patients with β-thalassemia as a result of cardiac iron overload contribute to 70% of cases [23]. Recent investigations have implicated IO in the dysregulation of autophagy and numerous disease states, and this will be discussed further below.

A study by Jahng *et al*. [24] indicated that the effect of IO on autophagy differed in a time-dependent manner. After 4-hr exposure to IO, cells exhibited LC3-II accumulation and p62 depletion, both of which are indicative of upregulated autophagy [24]. However, this upregulation was transient since prolonged IO led to autophagic flux inhibition. Reduced autophagy flux was indicated by the fact that a build-up of LC3-II was detected together with elevated p62 expression. These markers indicate that there was an accrual of autophagosomes and decreased turnover through lysosomal fusion and degradation [24]. Additionally, the authors demonstrated that reduced autophagy caused by prolonged IO exposure occurs due to reduced lysosome recycling, which is mediated by the autophagic lysosomal reformation (ALR) cycle. ALR requires mTORC1 reactivation through phosphorylation at S2448. However, prolonged IO can prevent reactivation of mTORC1 through AKT-mediated repression of TSC2 [24]. This finding was validated through recovery of mTORC1 phosphorylation by exposing cells to iron withdrawal. Likewise, in another experiment, it was found that prolonged IO exposure did not lead to decreased autophagic flux in Myc-RHEB GTPase mutant cells which maintain mTORC1 phosphorylation [24]. Although short term IO can upregulate autophagy, it is evident that prolonged IO has an inhibitory effect.

Iron homeostasis and autophagic flux display a reciprocal relationship. Autophagy mediates the release of free iron from ferritin, and this specific type of autophagy is termed ferritinophagy [25]. Delivering ferritin to lysosomes requires the cargo receptor, nuclear receptor coactivator 4 (NCOA4), which was identified by quantitative proteomics [26]. Under high intracellular iron conditions, NCOA4 can be downregulated upon its ubiquitination by HERC2, a ubiquitin ligase [27]. Ferritinophagy is stimulated during states of acute stress, such as pressure overload [25]. This process promotes intracellular ferrous IO, ultimately heightening lipid peroxidation and contributing to heart failure [25]. Indeed, in a study with mice that were subjected to pressure overload, it was found that the deletion of NCAO4 could attenuate IO and ultimately provide cardioprotective properties [25]. While it likely evolved as a protective mechanism, upregulated ferritinophagy is now implicated in the development of neurodegenerative diseases, urinary tract infections, and cardiac hypertrophy [28].

## 3. Oxidative Stress as a Mechanism Connecting IO and Autophagy

Metabolic processes in biological systems can generate reactive oxygen species (ROS) [29] and under physiological conditions, this generation of ROS is normal and can act as a signal transduction event [30]. However, under pathophysiological conditions, dysregulation and excess ROS production can occur. This oxidative stress is a well-established contributor to pathological conditions such as cancer, neurodegeneration, diabetes, and cardiovascular disease [31].

Due to its contribution to pathological conditions, oxidative stress is an attractive therapeutic target for the treatment of many disease states. For instance, numerous human clinical trials have studied antioxidants, to test their potential as treatments for diabetes and associated complications such as cardiovascular diseases [32, 33]. Unfortunately, it has generally been concluded that antioxidant therapies do not yield any significant benefit on cardiovascular diseases [32, 33]. The lack of therapeutic benefits has been attributed to reasons such as poor bioavailability and stability [34]. The use of antioxidants has resulted in reports of some unintended complications such as increased risk of bladder cancer and cardiovascular disease possibly due to inhibition of the physiological role of ROS or the pleiotropic nature of cellular events impacted by antioxidants [32, 33, 35, 36]. Nevertheless, the antioxidant catalase was reported to be protective against oxidative stress induced by cardiac dysfunction that was associated with AMPK-dependent autophagy [37]. Therefore, it can be suggested that therapies aimed at processes that are regulated by ROS may yield more effective treatment options. Autophagy is one such candidate since there is evidence suggesting crosstalk between oxidative stress and autophagy [38]. Autophagy can be activated or repressed in response to oxidative stress, whereas the inhibition of autophagy results in increased oxidative stress [38].

## 4. Oxidative Stress and Autophagy in the Heart

The regulation of autophagy and oxidative stress are both important for cardiac function [39]. The heart is particularly susceptible to changes in ROS levels and thus oxidative stress must be finely balanced [39]. Overall, autophagy is known to exhibit some protective effects against oxidative stress, while the loss of autophagy exacerbates it [37, 40].

Dysregulated autophagy plays a role in the pathogenesis of metabolic disorders as well. Insulin resistance is a condition where cells become less responsive to insulin and is an early indicator of type 2 diabetes [41]. It is widely accepted that oxidative stress contributes to the development of insulin resistance, however, the precise mechanism of this phenomenon remains to be fully defined [42]. Studies in cardiomyocytes have shown that autophagy plays a key role in oxidative stress-mediated insulin resistance. For example, in a study by Sung *et al*. [4], it was found that exposing cardiomyocytes to IO-induced oxidative stress led to insulin resistance and decreased autophagic flux. In this study, subsequent restoration of autophagic flux improved insulin sensitivity, which could indicate that the regulation of autophagy may play an important role in the development of diabetes. Thus, autophagy and oxidative stress both play important roles in determining the development of myocardial dysfunction. Given the lack of success with antioxidant treatments for oxidative stress associated diseases, acquiring a better understanding of the interactions between oxidative stress and autophagy may help facilitate the development of more effective therapeutic strategies. The interactions between oxidative stress and autophagy, as well as their implications on human health will be further discussed.

## 5. Mechanisms Linking Oxidative Stress and Autophagy

Studies in cardiomyocytes have shown that Forkhead box (Fox) transcription factors play a role in linking oxidative stress and autophagy. Under optimal growth conditions, upstream signaling pathways inhibit FoxO1 and FoxO3 activity through inhibitory phosphorylation. However, in conditions such as oxidative stress, these inhibitory signals are absent [43]. Oxidative stress is known to trigger a cellular protective response that involves the upregulation FoxO1 and FoxO3 activity [44]. This increased FoxO1 and FoxO3 activity results in increased activation of downstream gene targets involved in autophagy such as PINK1 and LC3-II, as well as antioxidants such as catalase and SOD2 [44]. As such, FoxO1 and FoxO3 act as part of a key defence mechanism against oxidative stress by facilitating the upregulation of autophagy genes and antioxidants. A mouse model study found that cardiomyocyte-specific loss of FoxO1 and FoxO3 resulted in increased myocardial injury and reduced antioxidant capacity following ischemia reperfusion injury [44]. Taken together, these findings suggest that oxidative stress upregulates FoxO1 and FoxO3, which in turn mitigates oxidative stress through downstream activation of antioxidants or autophagy as an adaptive defense mechanism. Nevertheless, the exact role of autophagy in resolving oxidative stress and attenuating adverse myocardial remodeling remains to be more fully investigated.

Although autophagy can promote improved cell survival after oxidative stress in cardiomyocytes, there is also evidence to suggest the contrary where autophagy can be detrimental. *In vivo* studies show that autophagy is upregulated following ischemia reperfusion injury, and this is associated with a greater area of myocardial injury [45]. However, beclin heterozygous knockout mice that were subjected to ischemia reperfusion injury exhibited a significant reduction in autophagy and size of myocardial injury [45]. Use of the antioxidant MPG attenuated oxidative stress-induced autophagy and resulted in reduction of myocardial injury in WT mice but not beclin heterozygous knockout mice [45]. Together, these results indicate that ischemia reperfusion induces oxidative stress, resulting in an increase in autophagy, followed by myocardial injury. The downregulation of autophagy alone was sufficient to reduce myocardial injury associated with ischemia reperfusion induced oxidative stress. This suggests that autophagy might be a better rate limiting factor and a highly appropriate therapeutic target.

**Fig. 2.**
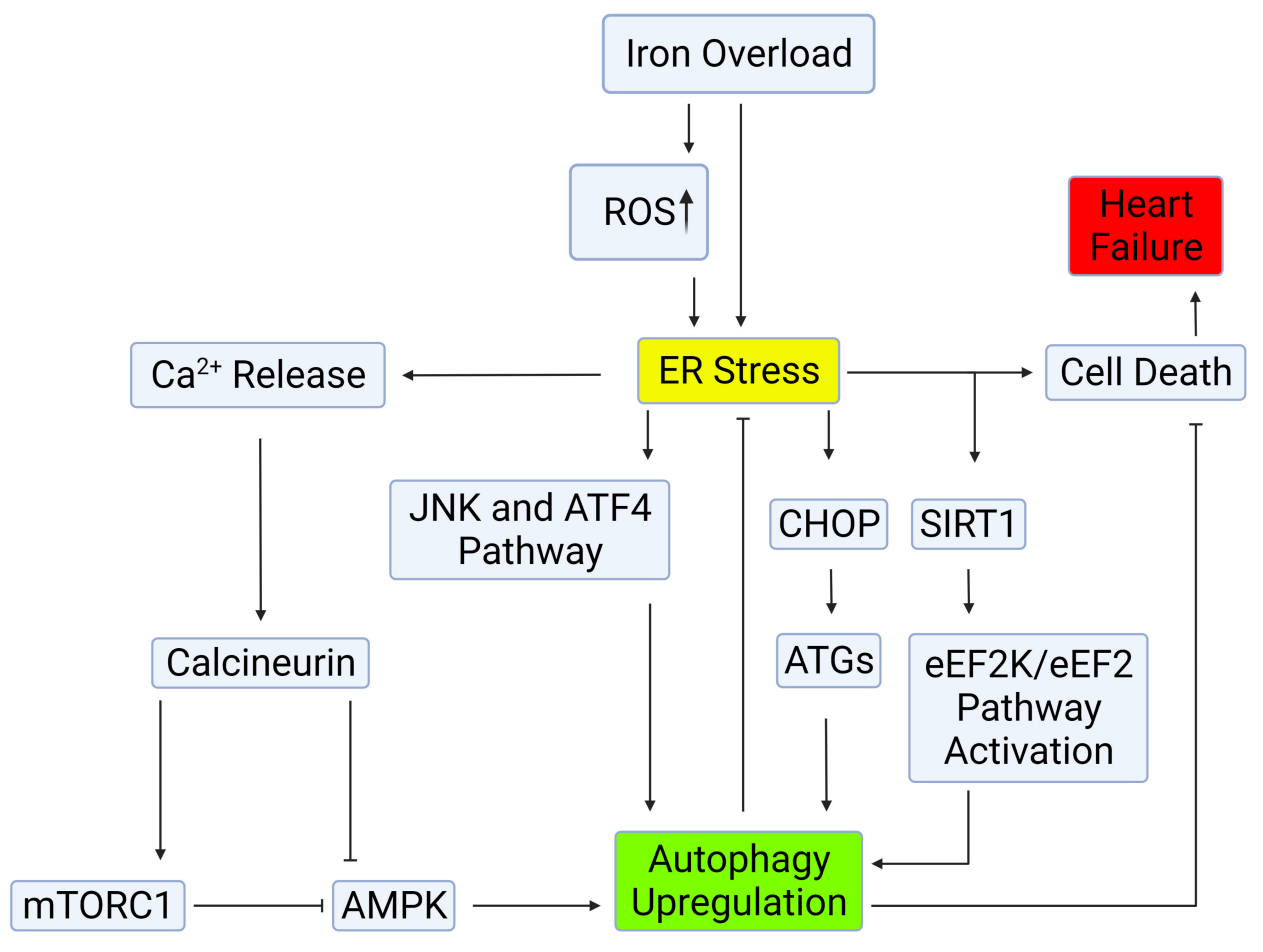
**Mechanisms of autophagy induction by ER stress**. Autophagy can be induced through the IRE-1–JNK/p38 or PERK-eIF2α-ATF4 pathways. ER stress mediates autophagy upregulation by calcium release, in which the CaMKKβ/AMPK pathway is involved in the inhibition of mTORC, consequently leading to autophagy upregulation. The deacetylase SIRT1 facilitates ER stress-induced autophagy through the activation of the eEF2K/eEF2 pathway, or through deacetylation of autophagy-related genes. Upregulation of autophagy upon ER stress hinders outcomes of cell death and exerts cardioprotective effects. Created with BioRender.com.

While it is known that oxidative stress can induce autophagy, there have also been reports that oxidative stress can inhibit autophagy. *In vitro* studies on cardiomyocytes showed an increase in autophagic flux following treatment with hydrogen peroxide [46]. LC3 expression increased following short-term exposure to hydrogen peroxide induced oxidative stress but declined after prolonged exposure [46]. AMPK phosphorylation at Thr172, its enzymatic active site, increased after oxidative stress, but decreased following long-term exposure [46]. Further investigation indicated that this regulation of autophagy was facilitated via calcineurin. Inhibition of calcineurin via FK506 relieved long term oxidative stress mediated inhibition of autophagy. Overexpression of calcineurin A (constitutively active form) inhibited autophagy during short term oxidative stress. Calcineurin was determined to be a key regulator of autophagy under oxidative stress where it can directly attenuate AMPK activity, thus decreasing autophagy as illustrated in Fig. 2 [46]. Therefore, modulation of autophagy by calcineurin inhibition could prove beneficial during oxidative stress. Genetic manipulation of AMPK confirmed calcineurin regulates AMPK activity, and consequently autophagy. It was concluded that short term oxidative stress promotes autophagy and long-term oxidative stress inhibits autophagy, akin to what was observed with IO.

## 6. Endoplasmic Reticulum (ER) Stress Contributes to Cardiomyopathy

The ER is responsible for various cellular functions, including protein synthesis, post-translational modification and trafficking, ${\mathrm{Ca}}^{2+}$ homeostasis, and lipid biosynthesis [47]. ER stress occurs upon altered cellular homeostasis in response to various pathological factors, such as nutrient deficiency, disrupted ${\mathrm{Ca}}^{2+}$ homeostasis, toxins and sustained oxidative stress. Importantly, cells respond to ER stress by initiating the unfolded protein response (UPR), a series of events designed to resolve ER stress and thus sustain cell viability and function. This includes activation of three transmembrane proteins: inositol-requiring enzyme 1 (IRE1), protein kinase RNA-like ER kinase (PERK) and activating transcription factor 6 (ATF6). These activated proteins aid in the restoration of cellular homeostasis by expanding the ER protein-folding capability, decreasing protein translation globally, and increasing ER-associated degradation (ERAD) [48]. Another important downstream effect of the UPR is induction of autophagy.

Studies have demonstrated that ER stress is implicated in the development and progression of heart disease. In heart failure patients with cardiac hypertrophy, expression of the major endoplasmic reticulum (ER) chaperone protein GRP78/BiP was increased along with markers of UPR activation such as splicing of XBP1 [49]. The Lys-Asp-Glu-Leu (KDEL), a retrieval receptor for ER chaperones in the early secretory pathway, is a contributing factor to ER quality control. Expression of a mutant KDEL *in vivo* resulted in expanded sarcoplasmic reticulum, protein aggregates, and enhanced expression of CHOP. This consequently led to the development of dilated cardiomyopathy. These findings suggested that impairment of the KDEL receptor resulted in an accumulation of misfolded proteins causing ER stress, and this was associated with dilated cardiomyopathy [50]. Furthermore, in rabbits left ventricular dilation, systolic dysfunction and cardiomyocyte apoptosis correlated with enhanced expression of GRP78 and CHOP [51]. In another study, overexpression of monocyte chemoattractant protein-1 (MCP-1) in mice with heart failure facilitated the proapoptotic effect of ER stress, resulting in greater myocardial damage [52]. The important role for turnover of intracellular proteins and organelles in the heart is well demonstrated in desmin-related cardiomyopathies (DRCMs), a group of disorders that originate from mutations in proteins such as αB-crystallin. Mutation of the gene coding for αB-crystallin creates a phenotype that includes protein aggregation, which may bring about ER stress and sudden cardiac death. Experiments have shown that blunting autophagy in mice harboring an αB-crystallin mutation considerably expedited both the progression of heart failure and mortality [53]. Finally, ER stress is considered a contributing factor to the pathogenesis of diabetic cardiomyopathy through induction of the endoplasmic reticulum stress response (ERSR) gene product, p53-upregulated modulator of apoptosis (PUMA) [54].

Taking these observations together, ER stress is strongly implicated in the development of heart failure and improved understanding of the molecular mechanisms via which UPR resolves ER stress, such as autophagy, will help better elucidate potential targets for drug discovery and novel therapeutic interventions.

## 7. ER Stress and Induction of Autophagy

Autophagy has been established as one effector mechanism via which UPR restores ER homeostasis [55]. Particularly upon prolonged ER stress and UPR activation, autophagy becomes a critical component of the adaptive response [56]. Autophagy can be induced through the IRE-1–JNK/p38 or PERK-eIF2α-ATF4 pathways in cardiomyocytes [57, 58]. The IRE-1–JNK/p38 pathway can upregulate beclin-1 by relieving inhibition of Bcl-2 or transcriptional upregulation [57]. The PERK-eIF2α-ATF4 can promote autophagy by transcriptional upregulation of autophagy-related genes (ATGs) [58]. ER stress also regulates autophagy by mediating ${\mathrm{Ca}}^{2+}$ release [59]. For instance, ER stress signals ${\mathrm{Ca}}^{2+}$ release from the ER lumen into the cytosol through the IP3R channel [59]. This process triggers the CaMKKβ/AMPK pathway and hinders mTORC1 activity, consequently facilitating autophagy induction [59]. In addition, the presence of ${\mathrm{Ca}}^{2+}$ activates death-associated protein kinase 1 (DAPK1), which causes phosphorylation of Bcl-2 and beclin-1, again leading to activation of autophagy [60]. It should be noted that although ${\mathrm{Ca}}^{2+}$ upregulates autophagy, it has also been shown to induce apoptosis highlighting autophagy’s dual nature [61]. Likewise, the deacetylase SIRT1 facilitates ER stress-induced autophagy through the activation of the eEF2K/eEF2 pathway, or through deacetylation of ATG5, ATG7 and LC3 [62, 63]. SIRT1 has also shown to exert cardioprotective effects in response to ER stress by eIF2α deacetylation, thus activating the PERK pathway [64]. Moreover, SIRT1 was found to play a role in activation of mitophagy upon ER stress by facilitating Parkin and LC3-II association with mitochondria [62]. The crosstalk between ER stress and autophagy induction is summarized in Fig. 2. Angiogenic factor with G patch and FHA domains 1 (AGGF1), which is crucial for vascular development, was found to be low in cases of heart failure [65]. AGGF1 also regulates ER stress through inhibition of ERK1/2 and downregulation of CHOP, resulting in prevention of ER stress-induced apoptosis [65]. However, it has been documented that CHOP can also upregulate ATGs in response to ER stress, thus acting as a pro-survival mechanism [66]. Thus, CHOP may play a key role in maintaining a balance between autophagy and apoptosis following ER stress. On the other hand, pre-activation of autophagy by ischemic preconditioning can activate defense mechanisms and reduce excessive ER stress [67]. Therefore, ER stress and autophagy are closely connected, and their interaction can play a role in determining the development or extent of heart failure.

Overload of misfolded proteins, such as polyglutamine repeat (polyQ)72 adducts in the ER, have been shown to trigger UPR activation, and consequently, autophagy. GRP78 and GRP94 protein levels are significantly elevated in ATG5-deficient hearts [8]. Activation of autophagy is a defense mechanism against polyQ72-induced ER-stress-mediated cell death, by degrading the protein aggregates and recruiting PERK/eIF2α phosphorylation in polyQ72-induced LC3 conversion [15]. Studies have found evidence that both eIF2α mutation and ATG5 deficiency in cardiomyocytes heighten the amount of polyQ72 aggregates and generate ER stress [68]. In these cases, activation of cleaved caspase-12 was increased, leading to ER-mediated cell death. Rapamycin, an autophagy stimulator, and 3-methyladenine (3MA), an inhibitor of autophagy, were used to determine the effects of autophagy on polyQ72-induced caspase-12 activation and DNA fragmentation. Results demonstrated that rapamycin lowered the amount of insoluble polyQ72 and obstructed caspase-12 activation (with a 50% decrease in the amount of the active form fragment), and DNA fragmentation. Meanwhile, 3MA escalated insoluble polyQ72 and triggered caspase-12 activation (with a twofold increase in the amount of the active form fragment), and DNA fragmentation [68]. Indeed, the downregulation of protein turnover could lead to overaccumulation of misfolded proteins, elevated endoplasmic reticulum stress, risk of apoptosis and cardiac dysfunction [8].

## 8. Impact of Autophagy on ER Stress

Conclusive demonstration on the functional significance of autophagy in regulating ER homeostasis was achieved in models with ablation of autophagy by conditional knockout of core ATG genes in cardiomyocytes. Knockout of ATG5 or ATG7 produces ER dilation, stress, inhibition of secretory protein transcription, cell death and inflammation [69]. Meanwhile, inhibition of autophagy reduced protective effects of Panax Notoginseng Saponins (PNS) against thapsigargin-induced ER stress [55]. This was supported by data in siATG7 transfected cells where the disruption and condensation of the ER tubular network were no longer affected by PNS pre-treatment [55]. No significant difference was observed in the expressions of BiP, CHOP, cleaved caspase-3, and caspase-12 in TG-induced cells, in the presence or absence of PNS pre-treatment [55]. Inhibition of autophagy allowed increased protein aggregate accumulation, as seen in CryABR120G-based desmin-related cardiomyopathy models [70]. Moreover, proteins that cannot be degraded by the ubiquitin-proteasome mechanism can be eliminated through the autophagy process. In doing so, autophagy simultaneously prevents ER stress and cell death that may have resulted from proteasome inhibition. As such, activated autophagy promotes survival, especially when the proteasome function is reduced [71]. These results imply that in cardiomyocytes, autophagy is required to facilitate protein quality control, and maintain cellular structure and function at physiological conditions [72]. Without autophagy, the accumulation of polyubiquitinated proteins may result in increased ER stress and, consequently, outcomes such as apoptosis.

## 9. Mitochondrial Dysfunction Induced by IO

Mitochondria play a significant role in iron metabolism as they sequester and utilize free iron for the synthesis of heme and iron-sulfur clusters. Excess labile iron in conditions of IO has a profound impact on ROS production, including from mitochondria [73]. The heart can be sensitized to such conditions of stress as mitochondria occupy an overwhelming 40–60% of cell volume within the myocardium and produce energy to meet roughly 90% of myocardial demands [74]. A sizeable perturbation in mitochondrial metabolism leads to the development of cardiomyopathy [73]. IO is one potential scenario in which this can occur. For instance, in an iron-overload mouse model (achieved via subcutaneous iron-dextran injection), cardiac hypertrophy and cardiomyopathy occurred, which was rescued following treatment with the iron chelator deferoxamine (1.5 mM/kg/week) [75]. Moreover, *in vitro* studies showed that prolonged IO suppressed mitochondrial enzymatic activities in rat cardiomyocytes, which were restored by iron chelation [76]. While iron exhibits a highly toxic effect on cardiomyocytes, the fact that this effect showed reversibility alludes to its therapeutic potential. A better understanding of how iron elicits myocardial damage has been developed via recent mechanistic studies and some of these are described below.

## 10. Impaired Autophagy and Mitochondrial Dysfunction

Mitochondria have been implicated as a node of crosstalk between oxidative stress and autophagy, and thus, can play a critical role in IO-induced autophagic dysfunction and subsequent heart failure. Treatment with palmitic acid (PA), an oxidative stress-inducing agent, upregulated intracellular and mitochondrial ROS, depolarized mitochondrial membranes, compromised mitochondrial morphology and, most notably, diminished autophagic flux in hepatocytes [77]. The addition of the bioflavonoid dihydromyricetin (DHM) restored mitochondrial membrane potential, oxidative stress and autophagic flux to normal [77]. Moreover, cardiotoxic effects have been identified in treatments of doxorubicin, a chemotherapeutic drug, in part through the induction of oxidative stress and impaired autophagic flux [78]. Doxorubicin also markedly upregulated mitochondrial fission, as well as cell death [78]. To assess whether doxorubicin’s effects on autophagy and mitochondria were indeed the culprits of its cardiotoxicity, another study tested donepezil, an acetylcholinesterase (AChE) inhibitor, to attenuate ROS and thereby rescue mitochondrial and autophagic dysfunction. This was based upon previous reports that found a correlation between elevated ROS and sympathetic overactivity. Moreover, sympathovagal imbalances were often observed in patients with myocardial infarction and heart failure [78]. In principle, to counteract sympathetic overactivity, donepezil would prevent degradation of acetylcholine (ACh) within the synaptic cleft, to drive sustained parasympathetic signalling and attenuate oxidative stress. Donepezil did indeed reduce systemic ROS via the reduction of sympathetic tone, resulting in the rescue of mitochondrial and autophagic functions and an alleviation of doxorubicin’s cardiotoxicity [78]. Together, these studies demonstrated the correlation between mitochondrial function and autophagic flux and their contribution to cardiomyopathy.

## 11. Mitophagy in Protection of Mitochondria against Oxidative Damage

**Fig. 3.**
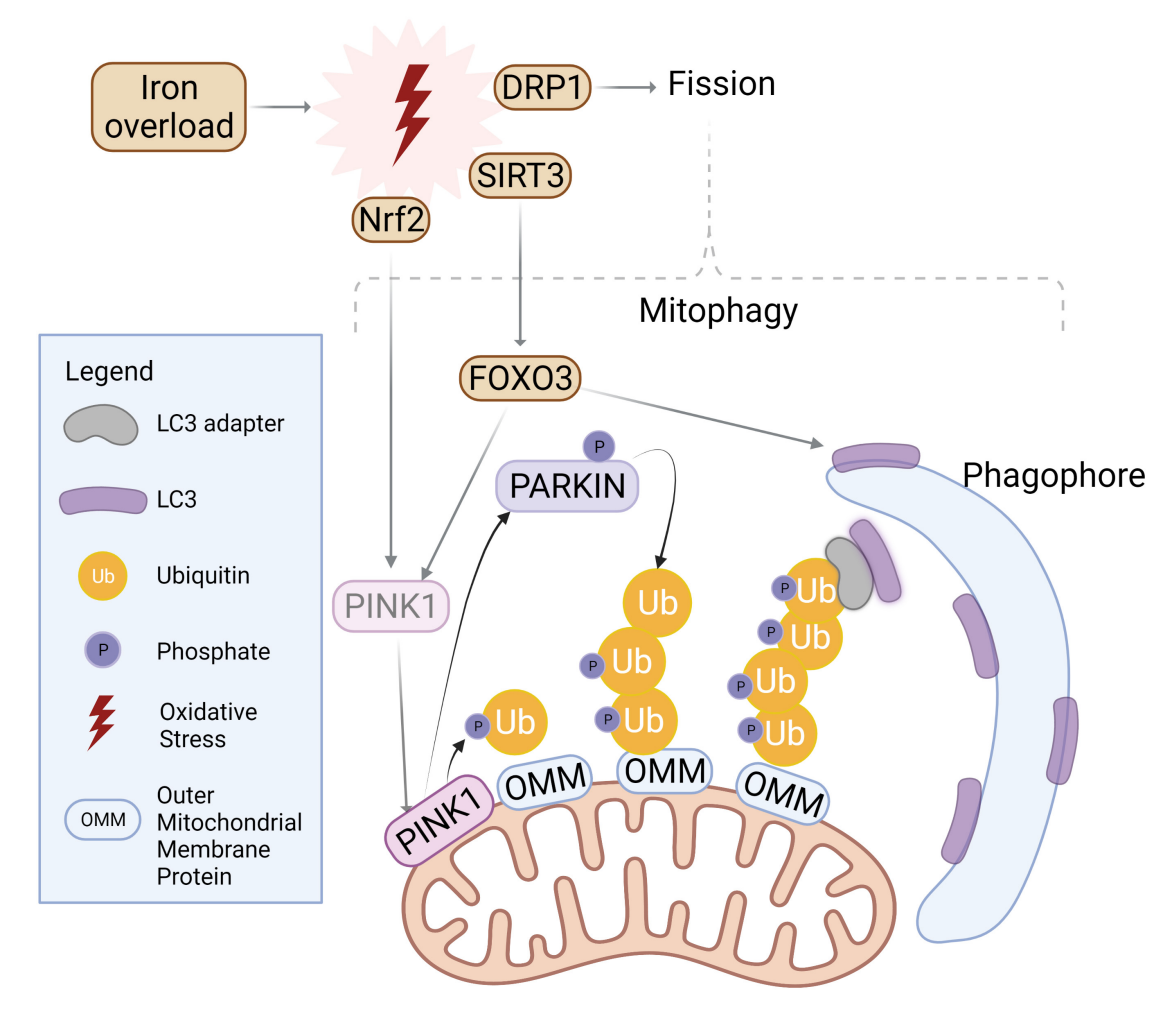
**Mitophagy induction by iron overload**. Oxidative stress incites nuclear localization and activation of transcription factor Nrf2 which upregulates PINK1. Simultaneously, SIRT3 is translocated from the nucleus to mitochondria in order to activate FOXO3, which upregulates PINK1 and LC3. PINK1 surrounds damaged mitochondria and phosphorylates Parkin for activation as well as ubiquitylated outer mitochondrial membrane (OMM) proteins. Parkin proceeds to polyubiquitinate OMM proteins while PINK1 follows suit in phosphorylating the ubiquitin chain. LC3 adaptors recognize this phospho-ubiquitin chain and recruits LC3 to initiate mitophagosome formation. Similarly, mitochondrial fission is upregulated upon oxidative stress and further incites mitophagy. Created with BioRender.com.

Autophagy is also an important contributor to maintenance of mitochondrial structure and function. To initiate mitophagy, PINK1 accumulates to selectively phosphorylate ubiquitin chain on the outer mitochondrial membrane, which in turn recruits Parkin for further polyubiquitination [79, 80]. LC3 adaptors such as p62, NDP52, or TAX1BP1 then recognize the ubiquitinated proteins and recruit LC3 with their LC3-interacting region (LIR), effectively trafficking mitochondria to the autophagosome [79] as summarized in Fig. 3. The balance between fission and fusion can determine the extent of mitophagy. Fusion of the outer mitochondrial membrane is carried out by mitofusin-1 and -2, whereas fission is mediated by dynamin-related protein-1 (Drp1) [81]. While Fis-1 is often cited as the mitochondrial receptor for Drp1, this interaction was specifically observed in yeast, whereas human Fis-1 appeared to mediate fission even in the absence of Drp1 through the obstruction of fusion machinery [82]. Fusion at the inner mitochondrial membrane is regulated by optic atrophy 1 (OPA1) [79].

Stimulation of fission through Fis-1 or Drp1 activation can often be driven by conditions of oxidative stress, where IO and ROS accumulation threatens the health of mitochondria, motivating mitochondria to split to mitigate damage [83]. Moreover, mitochondrial fission has been characterized as a prerequisite for mitophagy [84]. Knockout, or inhibition of Drp1 was shown to not only disrupt fission but also attenuate mitophagy, signifying the necessity for Drp1 to recognize damaged mitochondria and subsequently segregate them for degradation [85, 86].

It should also be kept in mind that although evidence clearly indicates that autophagy and mitophagy are connected, the two processes can function independently from one another [87]. Mild oxidative stress, which mimics elevated ROS upon physical exertion, was shown to induce mitophagy in a Drp1-dependent manner without triggering autophagy in mammalian cells [84], the details of which are summarized in Fig. 3. Oxidative stress affects the transcriptional activity of mitochondrial machinery. For example, the transcription factor Nrf2 is typically sequestered and selectively degraded within the cytosol [88]. However, upon conditions of oxidative stress, Nrf2 localizes to the nucleus to activate mitophagy-related genes such as PINK1, to counteract stress-induced cell death [88]. Instead, another target of oxidative stress, SIRT3, is transported from the nucleus to the mitochondria upon similar conditions to deacetylate and activate the transcription factor FOXO3, which then regulated expression of LC3 and PINK1 for mitophagy [89]. HIF-1α is another transcription factor that plays a role in regulating expression of mitophagy-related genes. Low oxygen levels increase ROS, which stabilizes HIF-1α, resulting in upregulated transcription of BNIP3 and NIX, which are known to promote mitophagy [90, 91, 92].

## 12. Exacerbating Mitochondrial Damage through Ferritinophagy

Another specialized form of autophagy termed ferritinophagy is iron-selective and has also been implicated in regulating the development of cardiomyopathy. Elevated ferritinophagy coincided with enhanced expression of sideroflexin1 (SFXN1), which traffics iron through the inner mitochondrial membrane [28]. The release of ferrous iron (${\mathrm{Fe}}^{2+}$) via elevated NCOA4-mediated ferritinophagy, followed by its subsequent trafficking into the mitochondria, was found to lead to mitochondrial IO and ROS overproduction in failing hearts [28]. This phenotype was rescued in mice through NCOA4 and SFXN1 gene silencing [28]. In summary, it is clear that mitochondria are an important node of crosstalk between disordered ROS and autophagic dysfunction.

## 13. Ferroptosis: An Iron Dependent form of Cell Death

Regulated cell death mechanisms such as apoptosis, necroptosis and pyroptosis play an important role in maintaining cellular homeostasis. On the other hand, dysregulated cell death can induce tissue damage and contribute to many disease states [93, 94, 95]. Although the term ferroptosis was first coined in 2012, this iron-dependent regulated cell death has been studied since the 1950s [96, 97]. Initially, ferroptosis was thought to be morphologically, biochemically, and genetically distinct from other types of cell death, such as apoptosis, necrosis and autophagic cell death [95]. However, evidence from recent studies indicates that there may be some similarities between ferroptosis and other types of cell death, particularly autophagic cell death. For example, evidence from RNAi screening and gene analysis studies suggests that multiple autophagy-related genes (ATG), including ATG 3 and ATG 13, are positive regulators of ferroptosis [98]. Nonetheless, cell death brought by intense iron accumulation and lipid peroxidation is unique to ferroptosis [95, 96, 99].

**Fig. 4.**
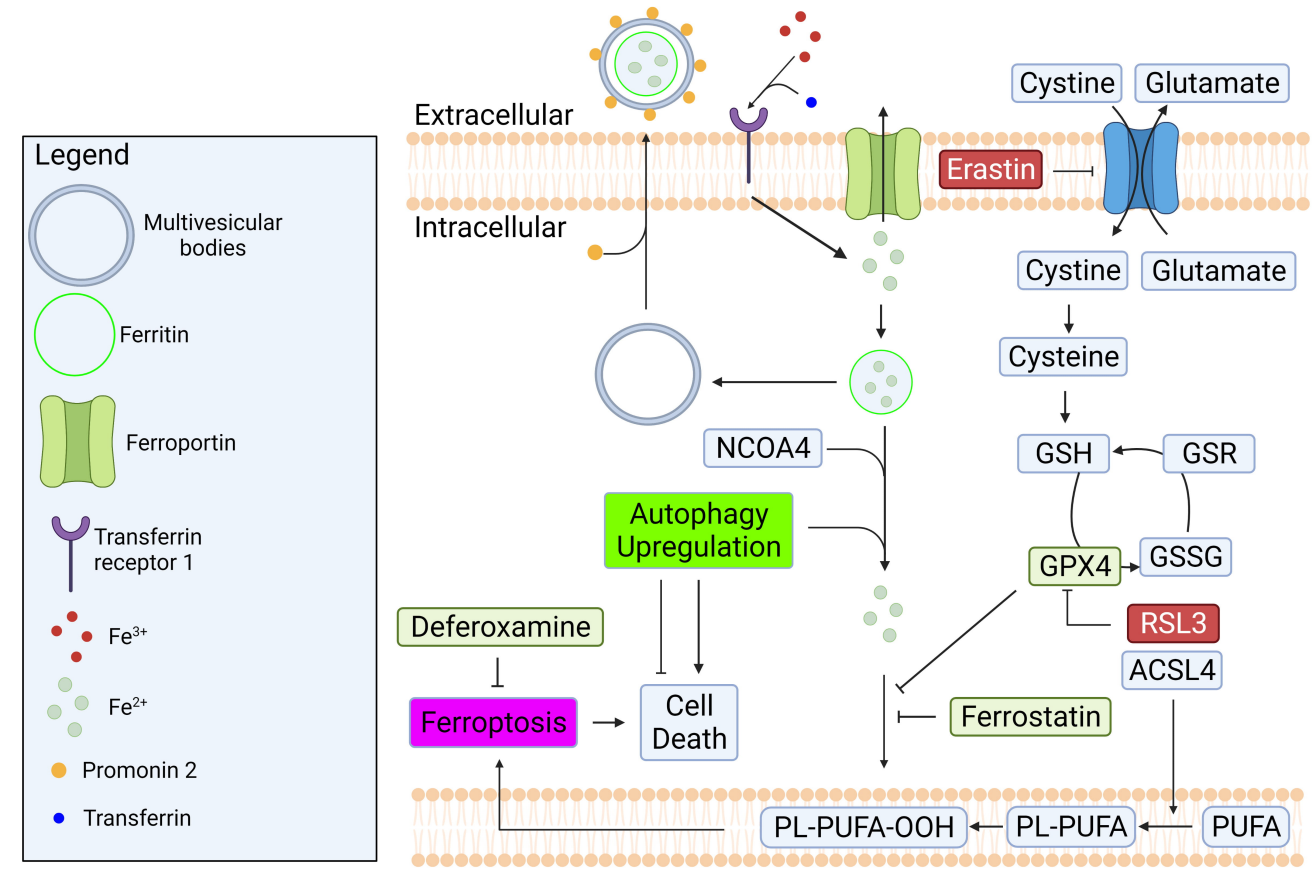
**Regulatory mechanisms of iron homeostasis and ferroptosis**. Iron homeostasis is regulated by balancing its import and export mechanisms. Excess iron causes lipid peroxidation which can lead to ferroptosis. GPX4 is a well-known enzyme that prevent ferroptosis by converting lipid hydroperoxides to non-toxic lipid alcohol. The inhibition of system ${\mathrm{Xc}}^{-}$ by erastin or GPX4 by RSL3 can cause ferroptosis. Created with BioRender.com.

In a healthy cell, intracellular iron concentration is kept at equilibrium through regular import and export mechanisms. To import iron into a cell, transferrin (a blood-plasma protein) first binds to ferric iron (${\mathrm{Fe}}^{3+}$) in circulation, then binds to transferrin receptor 1 (TfR1) on the cell membrane [100] as shown in Fig. 4. The binding of transferrin to TfR1 facilitates the endosomal importation of ferric iron (${\mathrm{Fe}}^{3+}$). While in endosomes, Fe3+ is converted into ferrous iron (${\mathrm{Fe}}^{2+}$) [101, 102]. After this conversion, ${\mathrm{Fe}}^{2+}$ is transported from the endoplasm and into the cytoplasm via a divalent metal transporter 1 (DMT1) [103]. In the cell, elevated levels of cytoplasmic ${\mathrm{Fe}}^{2+}$ could result in multiple different outcomes. Firstly, ${\mathrm{Fe}}^{2+}$ can be exported through the transmembrane protein ferroportin, to reduce intracellular ${\mathrm{Fe}}^{2+}$ concentration. Second, ${\mathrm{Fe}}^{2+}$ can bind to ferritin to be stored within the cell for future use. Ferritin is also the main iron cargo protein that can be exported in multivesicular bodies (MVBs) that are promoted by prominin 2 [104, 105, 106]. Lastly, ${\mathrm{Fe}}^{2+}$ can undergo oxidation through a Fenton reaction, which gives rise to lipid radicals [103]. Free radicals induce extensive oxidation of polyunsaturated fatty acid (PUFA)-containing phospholipids [103]. If oxidated (PUFA)-containing phospholipids integrate into the cell membrane, this can create instability in the membrane. TfR1 is considered an indirect marker for ferroptosis since high levels of TfR1 expression is correlated with abnormally high intracellular ${\mathrm{Fe}}^{2+}$ concentrations [100]. A high level of TfR1 may also act as an indicator of iron deficiency as the serum level of TfR1 has been used as a tool for the diagnosis of iron deficiency anemia [107].

Ferroptotic cell death can be brought on by an excessive accumulation of lipid reactive oxygen species (ROS) and lipid peroxidation by iron [95]. One of the core pathways modulating ferroptosis is system ${\mathrm{xc}}^{-}$/glutathione (GSH)/glutathione peroxidase 4 (GPX4) as illustrated in Fig. 4. The synthesis of antioxidant peptide GSH is initiated by importing cystine by system ${\mathrm{xc}}^{-}$, a membrane transporter that exchanges cystine and glutamate. After reduction of imported cystine to cysteine, ${\mathrm{xc}}^{-}$ catalyzes two consecutive steps with enzymes γ-glutamylcysteine synthetase and glutathione synthetase, both of which are required to activate GPX4 [108]. GPX4 is a phospholipid peroxidase that plays a central role in preventing ferroptosis by converting lipid hydroperoxides (L-OOH) to non-toxic lipid alcohols (L-OH) [109]. When GPX4 is inactivated through the depletion of glutathione with erastin, or through another direct GPX4 inhibitor like RSL3, the redox system becomes overwhelmed by lipid peroxidation. This condition ultimately leads to ferroptotic cell death [110]. Furthermore, the genetic deletion of GPX 4 or Slc7a11, a key subunit of system ${\mathrm{xc}}^{-}$, was shown to trigger ferroptotic cell death in mice [111, 112, 113].

Several enzymes of lipid metabolism are strongly implicated in ferroptosis. For example, it was found that the expression of acyl-CoA synthetase long-chain family member 4 (ACSL4), which catalyzes the conversion of fatty acid to form acyl-CoA, correlates with cellular sensitivity to ferroptosis induction [114]. For instance, when ACSL4 was suppressed by RNAi, cancer cells became more resistant to ferroptosis [114]. ACSL4 is known to increase lipid ROS through inhibition of GPx4 and biosynthesis of PUFA ω6 fatty acids for cellular membranes [115, 116, 117]. Ferroptosis is uniquely regulated by various mechanisms that involve the interplay between iron and lipid metabolism.

## 14. Interplay between Autophagy and Ferroptosis

Despite the important beneficial effects of autophagy, excessive levels can elicit autophagic cell death [118]. Many studies suggest that ferroptosis is a type of autophagy-dependent cell death [98, 119, 120, 121, 122]. As indicated above, ferritinophagy is a selective autophagic degradation process for the iron storage protein ferritin [118]. By sequestrating iron, ferritin plays an important role in regulating cellular iron levels and protecting cells from accumulation of labile iron. Degradation of ferritin by ferritinophagy releases chelated iron, which then becomes available as labile iron in lysosomal compartments and potentially the cytosol [120].

In previous studies, it was found that the genetic deletion of NCOA4 and ATGs (e.g., ATG3, ATG5, ATG7 and ATG13) could attenuate oxidative injury and ferroptosis by reducing ferritinophagy-mediated ferritin degradation, and by decreasing intracellular iron levels [98, 122]. These findings indicate that NCOA4-dependent autophagy (ferritinophagy) could be a positive regulator of ferroptosis. Moreover, several studies suggest that prolonged IO can lead to impairments in autophagic flux in cardiomyocytes and skeletal muscle cells, which contributes to insulin resistance [4, 24]. Other studies have shown that chronic IO conditions in the brain, heart and osteoblast cells can lead to increased autophagic flux in these cell types [123, 124]. Hence, effects of IO on various types of autophagy are dependent on factors such as time and cell type.

## 15. Potential Therapeutic Targets in Heart Failure

Ferroptosis is now established to be involved in many human diseases, including cancers, neurodegenerative diseases, metabolic diseases, and various forms of heart failure [125, 126, 127]. Many studies have suggested that ferroptosis could be a therapeutic target for cancer, through the modulation of targets such as NF2 (also known as merlin) -YAP signaling [125], the p62-Keap1-NRF2 pathway, the Ras/Raf/MEK pathway, and ferritinophagy [128, 129]. Ferroptosis has also been extensively studied and implicated in neurodegenerative diseases such as Parkinson’s and Alzheimer’s disease [126]. Of particular interest, studies on ferroptosis in non-alcoholic steatohepatitis (NASH) have found that ferroptosis plays an important role in exacerbating the disease. It was found that the pharmacological inhibition of ferroptosis by deferoxamine, lipostatin-1 and sodium selenite could alleviate disease progression in methionine-choline deficient diet-induced NASH mice [130, 131, 132].

Knocking out myocyte- and cardiomyocyte-specific ferritin H can lead to increased lipid peroxidation, reduce glutathione levels, and induce cardiac dysfunction with LV hypertrophy in high-iron diet-fed mice [133]. Numerous studies have indicated that ferroptosis occurs upon myocardial ischemia-reperfusion (I/R) injury [95, 134, 135]. In another model of cardiac injury using doxorubicin (DOX)-treated mice, there was upregulation of Heme oxygenase -1 (Hmox1) by Nrf-2, which led to an increase in systemic nonheme iron levels, and iron overload in mitochondria with concomitant mitochondrial lipid peroxidation. Notably, the inhibition of ferroptosis by ferrostatin-1 (Fer-1) in DOX-treated and cardiac I/R mice significantly attenuated ferroptosis-induced heart damage, reducing lipid peroxidation and infarct size, improving left ventricular (LV) systolic function and attenuating LV remodeling [127, 136]. The interplay with autophagy was more closely examined in a model of septic cardiomyopathy induced by administering lipopolysaccharide (LPS) to mice [137]. LPS caused an increase in lipid peroxidation, inflammatory markers, mitochondrial ROS, and cardiac dysfunction. Interestingly, when ferritinophagy was inhibited by silencing NCOA4, these consequences were attenuated [137].

Having described above some evidence implicating ferroptosis in heart failure and other prominent diseases, how can we intervene with appropriate therapeutic approaches? Iron can be imported into cells during IO via ${\mathrm{Ca}}^{2+}$ channels and the use of ${\mathrm{Ca}}^{2+}$ channel blockers such as verapamil and amlodipine could regulate cardiac iron accumulation and provide some protection against IO cardiomyopathy [138, 139]. Additionally, the use of iron chelators has been documented to have some success in preventing excess myocardial iron accumulation, yet iron chelation alone does not completely reverse the deleterious effects of iron overload [139, 140, 141]. As such, iron chelation in combination with complementary therapeutic strategies may prove to be more effective.

We described above the potential significance of IO-induced ER stress. The angiotensin-II blocker telmisartan is widely used to treat cardiovascular diseases [142, 143]. Telmisartan is now known to inhibit IRE1 and caspase 12, resulting in suppression of ER stress and ER stress-mediated apoptosis, respectively, and this may contribute to the overall beneficial effects [144]. Furthermore, telmisartan can alleviate metabolic dysfunction, a known risk factor for heart failure, through ER stress suppression [145]. Guanabenz is an inhibitor of Growth Arrest and DNA Damage-Inducible Protein (GADD34) and has been used as an antihypertensive drug [146]. Recent studies have demonstrated that guanabenz can also modulate the ER stress response by binding to and inhibiting protein phosphatase 1, thus sustaining eIF2α in its phosphorylated form to mimic UPR activation, resolving ER stress and conferring cardioprotective effects of guanabenz [147].

In conclusion, the recent rise to prominence of ferroptosis research has provided new knowledge on a mechanism via which iron overload can promote the development of cardiomyopathy. There is clearly crosstalk between ferroptosis and autophagy, already known to play a significant role in the pathophysiology of heart failure, although the nature of this relationship appears to be highly context dependent. Recent studies have also begun to indicate the therapeutic potential of targeting ferroptosis, and its interactions with autophagy, in the treatment of heart failure.

## 16. Conclusions

Autophagy is an important process that facilitates the clearance of damaged cytoplasmic contents to restore cellular homeostasis and dysregulation of autophagy has been implicated in several diseases. This review discussed the importance of autophagy in response to IO in the heart. The interactions between autophagy and other cellular processes such as oxidative stress, ER stress, mitochondrial health and ferroptosis were discussed.

In conclusion, IO causes ferroptosis, mitochondrial dysfunction, oxidative and ER stress. In response to IO, cells upregulate autophagy as a defense mechanism against the deleterious effects of IO. Without adequate upregulation of autophagy, IO causes cell death, ultimately contributing to heart failure. Nevertheless, it should also be borne in mind that an excess of autophagy or oxidative stress, ER stress, mitochondrial dysfunction and ferroptosis pose deleterious effects. Current knowledge of therapeutic targets related to IO, ER stress, ferroptosis, and heart failure were also discussed. We contend that therapeutic strategies targeting multiple cellular processes as discussed in this review may result in more beneficial results.
